# Genotypic Distribution and the Epidemiology of Multidrug Resistant Tuberculosis in Upper Northern Thailand

**DOI:** 10.3390/antibiotics11121733

**Published:** 2022-12-01

**Authors:** Sukanya Saikaew, Aksara Thongprachum, Rodjana Pongsararuk, Aungkana Thanraka, Naowarat Kunyanone, Boonchai Chaiyasirinroje, Praphan Luangsook, Bordin Butr-Indr, Ponrut Phunpae, Usanee Wattananandkul

**Affiliations:** 1 Faculty of Public Health, Chiang Mai University, Muang District, Chiang Mai 50200, Thailand; 2 Division of Clinical Microbiology, Department of Medical Technology, Faculty of Associated Medical Sciences, Chiang Mai University, Chiang Mai 50200, Thailand; 3 Office of Disease Prevention and Control, 1 (ODPC 1) Chiang Mai, Department of Disease Control, Ministry of Public Health Thailand, Chiang Mai 50000, Thailand; 4 Department of Medical Technology, Chiangrai Prachanukroh Hospital, Chiang Rai 57000, Thailand; 5 TB/HIV Research Foundation (THRF), Chiang Rai 57000, Thailand; 6 Infectious Diseases Research Unit (IDRU), Faculty of Associated Medical Sciences, Chiang Mai University, Chiang Mai 50200, Thailand; 7 Epidemiology Research Group of Infectious Disease (ERGID), Chiang Mai University, Chiang Mai 50200, Thailand

**Keywords:** rifampicin resistance, isoniazid resistance, mutation, tuberculosis, risk factor

## Abstract

The epidemiology and genotypes of multidrug-resistant tuberculosis (MDR-TB), a global public health threat, remain limited. The genotypic distribution and factors associated with MDR-TB in upper northern Thailand between 2015 and 2019 were investigated. The DNA sequencing of *rpoB*, *katG*, and *inhA* promoter of 51 multidrug-resistant *Mycobacterium tuberculosis* isolates revealed nine patterns of the *rpoB* gene mutation distributed in seven provinces. The S531L mutation was the most common mutation in all provinces. The *rpoB* mutation in Chiang Rai, Chiang Mai, and Lampang was highly diverse compared to other areas. Here, the mutation profiles that have yet to be reported in northern Thailand (H526P, Q513P, and H526C) were detected in Chiang Rai province. The S315T *katG* mutation was the most common genotype associated with INH resistance, especially in Chiang Mai and Lampang. Further analysis of data from 110 TB patients (42 MDR-TB and 68 drug-susceptible TB) revealed that <60 years of age was a significant factor associated with MDR-TB (OR = 0.316, 95% CI 0.128–0.784, *p* = 0.011) and ≥60 years of age was a significant factor associated with the S315T *katG*-mutation (OR = 8.867, 95% CI 0.981–80.177, *p* = 0.047). This study highlighted the necessity for continuous surveillance and risk factor monitoring for effective control of MDR-TB.

## 1. Introduction

Tuberculosis (TB) is an airborne infectious disease of global public health concern. The WHO estimates 1.6 million deaths from TB in 2021, which is an increase [1] from 1.4 million in 2019 [2] and 1.5 million in 2020 [3]. In 2021 there were 10.6 million TB cases worldwide of which 450,000 cases developed MDR/rifampicin-resistant TB (RR-TB). This is an increase compared to 9.9 million TB cases and 41,000 MRD/RR-TB cases reported in 2020 [1,3]. MDR-TB is caused by the *Mycobacterium tuberculosis* complex (MTBC) that is resistant to isoniazid and rifampin, the two most potent anti-TB drugs. The global cured rate of MDR/RR-TB in 2018 was only 59% [2]. Poor adherence to anti-TB therapy is the main cause of DR-TB, as well as treatment failure. Although most DR-TB develops by increased resistance to mutation (acquired resistance), the transmission of DR-strains from person to person (primary resistance) is possible through respiratory droplets. These cases have hampered global End TB strategies to reach the target of decreasing TB incidence, deaths, and catastrophic costs for TB-affected households at 95%, 90%, and 0% by 2035 [3].

Thailand was one of 12 high-burden countries (HBC) worldwide that was classified into two lists, TB incidence and TB with HIV (TB/HIV) by the WHO during the period of 2021–2025. It was estimated that there were approximately 103,000 cases of TB in Thailand in 2021. In addition, DR-TB cases doubled from 1200 in the year 2020 to 2400 cases in the year 2021 [1]. The top 5 provinces in Thailand with the highest incidence of TB include Bangkok, Samut Sakhon, Tak, Chiang Rai, and Chiang Mai. Two of the five provinces are in upper northern Thailand: Chiang Rai and Chiang Mai. In these provinces, the cure rate of TB was only 70.4%, which was lower than the 80% success target. In addition, TB incidence in the upper northern region (Chiang Mai, Chiang Rai, Lamphun, Mae Hong Son, Lampang, Phrae, Nan, and Phayao) has increased from 787 cases in 2017 to 1647 cases in 2018 [4]. This increase may be due to the problem of TB among migrant workers, especially those living in the North of the country. The frequent movement of immigrant workers, the inadequate treatment due to poor compliance and lack of resources, combined with tourists visiting from neighboring countries that have higher TB rates may also contribute to the increase in TB and DR-TB in this region [5]. According to a report from the Office of Foreign Workers Administration in 2015, 12,334 and 2801 migrant workers were working in Chiang Rai and Chiang Mai, respectively [6]. Without active surveillance and continued monitoring of the infection rate in this region, TB and MDR-TB could cause serious public health problems that are hard to control.

Rifampin (RIF) resistance is associated with the single point mutation of the *rpoB* gene encoding β-subunit of RNA polymerase of *M*. *tuberculosis* (Mtb). Among MDR-TB, more than 95% of the *rpoB* mutation occurs on the rifampicin resistance determining region (RRDR), an 81-bp fragment ranged from codon 432 to 458 in the *rpoB* gene of Mtb and codon 507 to 533 in the *Escherichia coli rpoB* gene [7]. As it has been reported in many countries, the most common *rpoB* mutation profile in Thailand is S531L causing the amino acid alteration from serine to leucine [8,9]. Unlike RIF resistance, isoniazid (INH) resistance is associated with the mutation of several genes, such as *katG*, *inhA*, *oxyR*-*ahpC*, and *ndh* [10]. Nevertheless, a single base change at codon 315 of the *katG* gene, which encodes catalase-peroxidase, is the most common mutation reported [10]. Globally, S315T causing an amino acid change from serine to threonine was found at a rate between 31% to 97% [11]. The second most common mutation associated with INH resistance occurs at −15 (C>T) within the promoter region of the *inhA* gene at the rate of 3.5% worldwide [12,13].

The variation in the rate of mutation in genes *rpoB*, *katG* and the *inhA* promoter with different profiles or positions appear depending on the geographic areas and the studied time period. Although *rpoB* mutation at codon 531 is the most common mutation worldwide, in some areas, for example, Zhejiang province, China, it was found that mutation at codon 526 was the most common [14]. In Thailand, a study in 2010 reported the most common *katG* mutation profile were S315T and R463L [15]. Recently, Anukool et al., 2020 reported S315T in *katG* mutation at 46.7% and −15 (C>T) in *inhA* promoter at 13.3% of 30 MDR-TB isolates from northern Thailand [9]. However, the current information on the mutation profiles and the distribution of MDR-TB genotypes in each province in northern Thailand is still lacking. Little is known about the factors that may account for MDR-TB in this area. Moreover, there were no data on the relationship between the MDR-TB epidemiology and mutation profiles. Hence, this study aims to describe the evidence of genotypes, prevalence, and distribution of MDR-TB in upper northern Thailand and examine the factors that may be associated with MDR-TB in this region. The evidence-based knowledge gained from this study may be useful for further establishment of an appropriate intervention for the control of MDR-TB.

## 2. Results

### 2.1. The Genotypic Distribution of MDR-TB in Upper Northern THAILAND

Among 51 MDR-Mtb isolates, the most frequent mutation found in the *rpoB* gene was codon 531 (26 isolates, 50.94%). Mutation at codon 526, 516, 522 and 513 was found in 19 isolates (37.25%), 3 isolates (5.88%), 1 isolate (1.96%), and 1 isolate (1.96%), respectively. Mutation in the *rpoB* gene was not found in 1 isolate (1.96%). The *rpoB* gene mutation in all isolates was characterized into 9 profiles including S531L (50.98%), H526Y (19.61%), H526D (11.76%), D516V (5.88%), S522L (1.96%), H526R (1.96%), H526P (1.96%), Q513P (1.96%) and H526C (1.96%) (Table 1). Three mutation profiles, H526P, Q513P, and H526C were initially reported in upper northern Thailand. The mutation of the *katG* gene and *inhA* promoter was found in 42 of 51 MDR-Mtb isolates (82.35%). Only one mutation profile of the *katG* gene, S315T, was found in 27 isolates (52.94%). Four mutation profiles of the *inhA* promoter were found in 15 isolates (29.41%) including −17 (G>T) (3.92%), −8 (T>G) (5.88%), −15 (C>T) (15.69%) and −9 (T>C) (1.96%). The *inhA* promoter mutation profiles that were found in conjunction with S315T *katG* mutation included −15 (C>T), −17 (G>T), and −8 (T>G) (Table 1). However, the majority of MDR-Mtb isolates harbored the −15 (C>T) mutation profile as a single mutation. Moreover, 9 MDR-Mtb isolates (17.65%) with no mutation in both the *katG* gene and *inhA* promoter were found.

**Figure 1:**
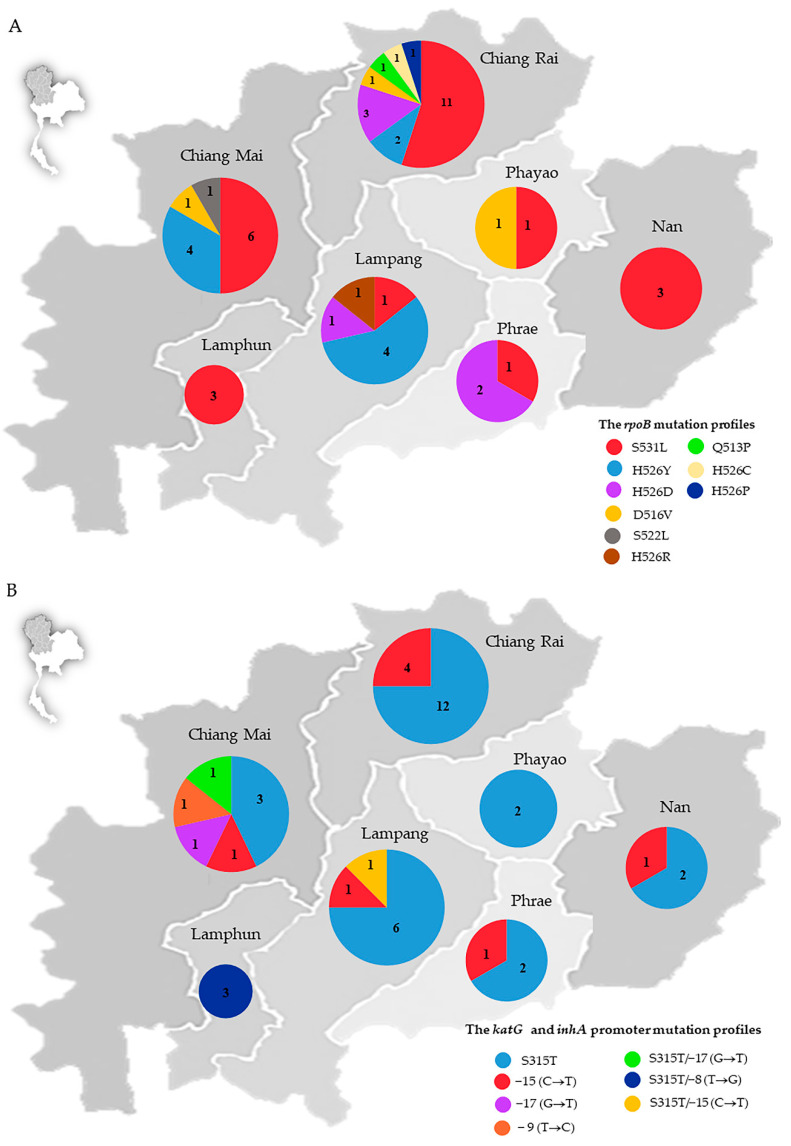
The distribution of multidrug-resistant *Mycobacterium tuberculosis* genotypes in seven provinces in upper northern Thailand: (**A**) the *rpoB* gene mutation profiles and (**B**) the *katG* gene and *inhA* promoter mutation profiles.

The genotypic distribution of mutation in the *rpoB* gene (Figure 1A) and *katG* gene and the *inhA* promoter (Figure 1B) of 51 MDR-Mtb isolates in seven provinces in upper northern Thailand (Chiang Mai, Chiang Rai, Lamphun, Lampang, Phayao, Phrae and Nan) was determined. The S531L MDR-TB was the most common *rpoB* mutation distributed in all provinces. The genotypes of the *rpoB* gene in MDR-Mtb found in Chiang Rai, Chiang Mai, and Lampang were highly diverse compared to other areas. Only one *rpoB* mutation profile (S531L) was detected in Lamphun and Nan. In Phayao and Phrae, there were two *rpoB* mutation profiles found. In addition, the mutation profiles that have never been reported in northern Thailand (H526P, Q513P, and H526C) were detected in Chiang Rai province (Figure 1A).

Among the mutations found in the *katG* gene and the *inhA* promoter, the S315T mutation in the *katG* gene was the most common (Figure 1A). It was found distributed in all provinces. A high mutation diversity in the *katG* gene and the *inhA* promoter appeared in Chiang Mai and Lampang provinces. Only one mutation profile was found in Lamphun and Phayao. Two mutation profiles were detected in Chiang Rai, Nan, and Phrae. The double mutation of the *katG* gene and the *inhA* promoter was found in Chiang Mai, Lampang, and Lamphun provinces (Figure 1B).

The frequency of different genotypes of 51 MDR-Mtb isolates in upper northern Thailand is shown in Table 2. The most frequently found genotype was MDR-Mtb with S531L-*rpoB*/S315T-*katG*/*inhA* promoter non-mutation (*n* = 11, 21.57%). The second most detected MDR-Mtb strains were containing S531L-*rpoB*/−15 (C>T)-*inhA* mutation/*katG* non-mutation and H526Y-*rpoB*/S315T-*katG*/*inhA* promoter non-mutation (*n* = 6, 11.76%). There were five isolates (9.80%) containing S531L-*rpoB*/*katG* non-mutation/*inhA* promoter non-mutation and H526D-*rpoB*/ S315T-*katG*/*inhA* promoter non-mutation. The S315T-*katG* was found in a combination of all *rpoB* mutation profiles except Q513P-*rpoB*. In contrast, the −15 C>T *inhA* promoter genotypes were found together with four *rpoB* mutation profiles (S531L, H526Y, H526D, and H526R). Only a single MDR-Mtb isolate contained *rpoB* non-mutation/ S315T-*katG*/*inhA* promoter non-mutation (Table 2).

### 2.2. Demographics and Characteristics of Tuberculosis in Upper Northern THAILAND

A total of 110 cases of TB, 42 cases of MDR-TB and 68 cases of drug-susceptible TB (DS-TB) were analyzed in this study. The majority of patients were male (*n* = 78, 70.91%). Most patients were Thai (*n* = 102, 92.73%), while four cases were from Myanmar, and in four cases the nationality was unspecified. The average age of 110 TB patients was 49.85 ± 17.69 ranging from 14 to 92 years old. The average age of DS-TB was higher than that of MDR-TB (52.95 ± 17.38 vs. 48.3 ± 15.6). The proportion of patients aged <60 years old in MDR-TB was higher than that of DS-TB (80.95% vs. 57.35%). However, the number of patients aged ≥ 60 years old was found lower than MDR-TB (19.05% vs. 42.65%). The statistical analysis also showed a significant association between the patient’s age and MDR-TB (OR = 0.316, 95% CI 0.128–0.784, *p* = 0.011) (Table 3). The proportion of males with MDR-TB was lower than that of DS-TB (64.29% vs. 75.0%), while the proportion of females was relatively similar in MDR-TB and DS-TB (35.71% vs. 25.0%). However, the association between gender and MDR-TB was not statistically significant (*p* = 0.229). In addition, other factors, such as nationality, province of residence, AFB smear result, and DST, were not significantly associated with MDR-TB in upper northern Thailand (Table 3).

### 2.3. The Association of MDR-TB and the Mutation Profiles in rpoB, katG, and the inhA Promoter

The association of MDR-TB patient data and a key mutation profile related to RIF resistance was determined using data from 42 MDR-TB patients: 22 MDR-TB cases with S531L-*rpoB* mutation (S531L MDR-TB) and 20 MDR-TB cases with other mutation profiles of the *rpoB* gene (non-S531L MDR-TB). The result showed that the majority of S531L MDR-TB cases were male (77.27%), but the same proportion of females and males in non-S531L MDR-TB were found (50%) (Table 4). However, the statistical analysis showed no significant association between gender and S531L MDR-TB (*p* = 0.107). The average age of S531L MDR-TB was lower than that of non-S531L MDR-TB patients (44.8 ± 13.9 vs. 52.2 ± 16.8). A higher number of patients aged ≥ 60 years old was observed in non-S531L MDR-TB compared to S531L MDR-TB (30% vs. 9.09%). However, patient’s age was not significantly associated with S531L MDR-TB (*p* = 0.023) (Table 4). In addition, nationality, province of residence and results from AFB smear and DST were not significantly associated with the *rpoB* mutation profiles (*p* > 0.05) (Table 4).

To study the association between MDR-TB and a key mutation profile related to INH resistance, data from 22 MDR-TB cases with S315T-*katG* mutation (S315T MDR-TB) and 20 MDR-TB cases containing other mutation profiles of the *katG* and the *inhA* promoter gene (non-S315T MDR-TB) were analyzed. There was no significant association of S5315T MDR-TB with gender, nationality, province of residence, or results from AFB smear and DST (*p* > 0.05). The average age of S5315T MDR-TB cases was higher than that of non-S315T MDR-TB patients (54.3 ± 16.4 vs. 41.7 ± 11.9). However, a higher number of patients aged ≥60 years old was observed in S315T MDR-TB (31.81%) compared to non-S315T MDR-TB. The statistical analysis also showed a significant association between the patient’s age with S315T MDR-TB (OR = 8.867, 95% CI 0.981–80.177, *p* = 0.023) (Table 5).

## 3. Discussion

In this study, we examined the genotypes of MDR-Mtb isolates in upper northern Thailand. It was found that approximately 98% of MDR-Mtb isolates harbored mutations within the RRDR in the *rpoB* gene. This is consistent with reports from other studies that approximately 90–95% of RIF-resistant Mtb (RR-Mtb) contains a point mutation within the 81-bp hotspot region (codons 507 to 533) of the *rpoB* gene [16,17]. Here, we found that codon 531 was the most common mutation found in the *rpoB* gene among 51 MDR-Mtb isolates (50.98%). The second most common mutation was found at codon 526 (37.25%), whereas the mutations at codons 516, 522, and 513 were detected in a few Mtb isolates. These findings correspond to most studies in Thailand [8,9,18]. However, of the *rpoB* mutation profiles detected, the prevalence of each mutation profile varied depending on the time and geographic location of the study. Prammananan et al., 2008 reported that 58% of MDR-Mtb isolates (*n* = 143) collected during the years 2003–2005 from 53 provinces (representing all regions in Thailand) had a mutation at codon 531 [8]. However, a 2017 report investigating the genotypic distribution of MDR-Mtb, pre-XDR-Mtb and XDR-Mtb (*n* = 34) in northern Thailand found that *rpoB* mutation at codons 588 and 589 (*Escherichia coli* codon) was the most common during 2005–2012 (26.5%) [18]. The second most common mutation was found at codon 571 (20.6%), followed by codon 531 (17.1%) [18]. The studies of *rpoB* mutation in 90 RR-Mtb isolates (most of them from Asian countries) also showed mutation at codon 531 (56.5%) was dominant [19]. Additionally, DNA sequencing results of 72 MDR-Mtb isolates confirmed that the mutation at codon 531 was the most common in China (41.0%), followed by codon 526 (40.0%) [20].

In this study, the *rpoB* mutation in 51 MDR-Mtb isolates was characterized. Nine profiles were discovered and the *rpoB* mutation was not found in one isolate (1.96%). The S531L mutation profile was the most common mutation (50.98%). The results are consistent with previous studies in Thailand [8,9]. A recent study on the genotypic distribution of MDR-TB in northern Thailand reported six mutation profiles: S531L (50%), H526Y (26.68%), H526D (10%), D516V (3.3%), S522L (3.3%), and H526R (3.3%) [8]. The S531L mutation accounted for 57.75% of MDR-TB in Thailand [8]. Additionally, this study detected H526P, Q513P, and H526C, which have not been reported in northern Thailand. The MDR-Mtb harboring these mutations was isolated in Chiang Rai province (Table S1). Two isolates, H526P and Q513P mutant strains, were positive for the AFB smear but an isolate harboring the H526C mutation was AFB negative. The DST results indicated that all three strains were sensitive to both ethambutol and streptomycin. Although they have not yet been detected in the northern region, these three mutation profiles have been reported elsewhere in Thailand and in other countries. The H526C mutation profile was found in one RR-Mtb isolate from the Central Chest Institute of Thailand. However, this RR-Mtb isolate contained a double mutation, in which both H526C and L533R mutations were found within the *rpoB* gene of an isolate [21]. Therefore, this study is the first report of a single mutation of the H526C mutation in Thailand. An investigation of MDR-TB genotypes in Thai patients revealed that the Q513P mutation was found in one isolate of 143 MDR-Mtb, isolated in 53 provinces (0.70%) [8]. The detection of the H526P mutation was reported in one of 50 MDR-Mtb isolates (2%) by a study at ODPC 5, Ratchaburi province, western Thailand [22]. The DST results indicated that this H526P-*rpoB* mutant isolate was resistant to pyrazinamide (PZA) but no mutation within the *pncA* gene was detected by DNA sequencing method. The authors discussed that other genes, such as *rpsA*, and the decrease of drug influx or the increase of drug efflux could be involved in PZA resistance in this isolate [22]. However, the susceptibility to PZA of the H526P–mutant isolate found in our study, was not performed.

The mutation profiles H526P, H526C, and Q513P were also reported in a study in the South-central region of China. Each was found in a single isolate among 60 RR-Mtb isolates collected from patients admitted to Wuhan Medical Center, Wuhan, Hubei [23]. The study reported that the H526P and H526C Mtb isolates had a single-point mutation. A high RIF MIC of the H526P-mutated strain was observed at >8 µg mL^−1^, whereas the RIF MIC of the H526C-mutated isolate was 2 µg mL^−1^. A strain that carried the Q513P mutation had the double point mutation in the *rpoB* gene, Q513P and E458A mutation. This isolate showed a high RIF MIC of >8 µg mL^−1^ [24]. The correlation between RIF MIC and the RRDR mutation was also investigated in MDR-Mtb isolates from California, India, the Philippines and South Africa [25]. They found that two isolates harboring H526C showed different RIF MIC: 2 µg mL^−1^ in one isolate and 8 µg mL^−1^ in another. A high RIF MIC of >8 µg mL^−1^ was observed in the Q513P-mutated strain. In addition, recent research in China showed a considerably high RIF MIC of ≥128 µg mL^−1^ in one Q513P-mutated isolate, though an isolate with H526C-*rpoB* mutation showed a RIF MIC of 2 µg mL^−1^ [25]. These results suggest the association of H526Pand Q513P *rpoB* mutations with the elevated RIF MIC (>8 to ≥128 µg mL^−1^) in Mtb strains. However, these mutation profiles were rarely found and were reported only in certain regions including Asia and South Africa [22,23,24,25].

The genomic studies of both clinical isolates and model organisms have revealed that resistance to RIF is strongly associated with the selection of fitness-compensating mutations in the different subunits of RNA polymerase [26]. In this study, the Q513P, H526C and H526P mutations were found to be rare in upper northern Thailand, implying a high fitness cost and less competitiveness among these strains. Compared to DS-Mtb and the strains carrying the mutation with a lower fitness cost, Mtb strains with these mutation profiles are unlikely to survive if they are in non-antimicrobial environment. Hence, they are less common and poorly distributed. However, the fitness cost of these three mutated Mtb strains has not been reported. Moreover, we found that one of the 51 MDR-Mtb isolates contained no mutation within the *rpoB* gene. This strain was also found in several studies and in our previous work [9,18]. Other mechanisms, such as the efflux pump mechanism, a reduction in drug delivery to cells, and changes in the drug binding targets may be involved in RIF resistance [27,28]. Despite the mutation within the RRDR, the mutation could occur outside the 543-bp sequenced region of the *rpoB* gene [20].

Only one mutation profile of the *katG* gene, S315T was found at 52.94% of all MDR-Mtb isolates, but four mutation profiles of the *inhA* promoter (−8, −9, −15, and −17) were detected at 29.41%, the most being −15 (15.67%), were found. The *inhA* promoter mutation profiles that were found in conjunction with S315T *katG* mutation were found at 9.8%. Up to 17.65% of MDR-Mtb isolates contained no mutation in both the *katG* gene and *inhA* promoter. However, a lower rate of S315T *katG* mutation was found in a previous report in 2017 in Thailand’s northern region (35.3%, *n* ***=*** 34) [18]. They reported the most common *inhA* gene mutations at codons 14 (32.4%) and 114 (32.4%) in Mtb isolates. The results are inconsistent with this study; the most frequent mutation profile was found as −15 within the *inhA* promoter region. This is consistent with a previous report in northern Thailand in 2020 [8]. The study reported S315T *katG* mutation at 63.3% of 30 MDR-Mtb isolates and the four mutation profiles with the same positions were discovered at the upstream of *inhA* (−8, −9, −15, and −17). The mutation of the *inhA* promoter at −15 (C>T) was found predominant in both this study and the previous study in northern Thailand [9]. The study of more MDR-TB isolates confirmed the prevalence of different mutation profiles in the *katG* gene and the *inhA* promoter in this area. However, a higher number of MDR-Mtb isolates along with continuous monitoring of MDR-TB genotypes is needed. The genotypic analysis of genes associated with INH resistance in 160 MDR-TB isolates in Thailand in 2005–2006 found that mutations in the *katG* gene accounted for 80.6% (*n* = 129). The S315T *katG* mutation was predominantly found at 80.0% (*n* = 128). Nine patterns of the single point mutations of the *inhA* gene and *inhA* promoter were found [−15 (*n* = 13), S94A (*n* = 2), −8 (*n* = 1), −15/G40W (*n* = 1), −15/I21N (*n* = 1), −15/I21V (*n* = 1), −15/S94A (*n* = 1), S94W, L11V (*n* = 1)]. The most common mutation was found at −15 of the *inhA* promoter with a rate of 10.63% (*n* = 17) [15]. The mutation pattern within the *inhA* gene was also analyzed, and the mutation at four codons; codons 11, 21, 40, and 94, was delineated. Here, the mutation within the promoter region of the *inhA* gene was investigated. Further genotypic analysis within the *inhA* gene may provide more information on the mutation profiles related to INH resistance in MDR-Mtb isolates in upper northern Thailand.

In 2010, a study in the Republic of South Korea reported that the single profile of the *katG* gene mutation, S315T, was found predominate at 57% (*n* = *57*) of 100 INH-R Mtb isolates. This is consistent with our work that found the S315T mutation at 52.4%. The single mutation profile of the *inhA* promoter, −15 C>T, was found at 22% (*n* = 22) of all INH-R Mtb isolates. The double mutations (both *katG* and *inhA* were mutated) were detected as S315T/−15 C>T (10%) and D310A/−15 C>T (1%) [29]. However, our study detected several mutation profiles of the *inhA* promoter. In addition, three profiles of double mutation: S315/−8, S315T/−15, and S315T/−17 were reported here. In 2016, a study investigated mutation profiles in the *katG* genes and the *inhA* promoter in MDR-Mtb isolates from Peru (*n* = 78). The S315T mutation profile was reported as the only mutation profile detected within the *katG* gene. It was found at 84.6% (*n* = 66), which is higher than the rate of S315T found in this study. Only a −15 C>T mutation profile was detected within the *inhA* promoter (14.10%, *n* = 11). A double mutation (S315T/−15) was found in one isolate (1.3%) [30]. Nevertheless, the rate of −15 C>T and S315T/−15 C>T was found to be lower compared to our study u-Mtb isolates (18%) that are concordant with our study, where 17.67% of non- *katG*/*inhA* mutated isolates were found. However, previous investigations showed the rates of MDR-Mtb containing no mutation within the *katG* and *inhA* promoter ranging from 16–46% [9,12,18]. The INH resistance in these isolates may be due to mutations within the *inhA* gene (not at the promoter region) [15] and other genes reported to be associated with INH resistance in Mtb, such as *OxyR*-*ahpC*, *emb*, *kasA*, and *ndh*  [9]. Lately, a study demonstrated the relevance and utility of whole genome sequencing (WGS) as a high-resolution approach to perform a genetic analysis of drug-resistant TB [31]. WGS applies next-generation sequencing, and therefore might be the most reliable solution to detect and characterize all mutations or genes associated with the resistance to INH and other anti-TB drugs that could not be identified by a conventional DNA sequencing method.

The genotypes of MDR-Mtb found in Chiang Rai were highly diverse. The *rpoB* mutation profiles that have never been reported in northern Thailand (H526P, Q513P, and H526C) were detected in this province. There were seven patterns of *rpoB* mutations, and two mutation patterns in the *katG* gene and the *inhA* promoter in Chiang Rai. In Chiang Mai, only four patterns of *rpoB* substitution were found, but there were five patterns of the *katG* gene and *inhA* promoter mutation. The variation of mutation profiles found in other provinces was less than that found in the two provinces. However, the number of MDR-Mtb isolates studied was fewer in these provinces compared to Chiang Mai and Chiang Rai. These two provinces are the two most densely-populated provinces in upper northern Thailand. While Chiang Rai is the northernmost major city in Thailand, it is bordered by Myanmar to the north and Laos PDR to the east. Chiang Mai is the second largest city in Thailand and the capital of Thailand’s northern region. The population of the two biggest cities has better access to the public health care system, including TB treatment. However, patient nationality and province of residence were not significantly associated with MDR-TB cases. In addition, other factors, such as treatment compliance, history of TB, underlying disease, HIV co-infection, and socioeconomic conditions were not available for statistical analysis.

The results of the demographic characteristics of TB and MDR-TB in upper northern Thailand showed that the majority of patients were male (75% and 64.29%). This is consistent with other reports in Thailand. The results from a study of 290 pulmonary TB patients at the Central Chest Institute of Thailand (CCIT) showed that 65.5% of TB and MDR-TB were male [32]. A case-control study of patients clinically diagnosed and microbiologically confirmed to have TB at Siriraj Hospital, Bangkok, from 2010 to 2012, found that 57.4% of 118 patients were male [33]. The predominance of men in tuberculosis has been reported elsewhere [34,35,36,37,38], however, the results from this investigation showed that gender and other factors, such as nationality, province of residence, and AFB smear and DST results, have no association with MDR-TB. These results are similar to previous research. [32,33]. In Thailand, a study of 290 confirmed TB cases (MDR-TB = 145; DS-TB= 145) at the CCIT between January 2007 and December 2013 found that the baseline characteristics of age, gender, residential area, education, and underlying medical illnesses were not significantly linked to MDR-TB. Nevertheless, investigators found that prior pulmonary TB management with a non-category I regimen, treatment failure or default to treatment outcomes, and particular characteristics of lung cavities, were significant risk factors of MDR-TB [32]. Another study at Siriraj Hospital investigated the risk factors of MDR-TB among 1118 TB cases (MDR-TB = 47; non-MDR-TB = 141) from 2010 to 2012. The results show no significance in gender, BMI, TB contact, underlying disease, type of infection or positive AFB smear [33]. However, it was found that MDR-TB is significantly associated with: patients under 65 years old, a previous history of TB, HIV co-infection, and alcohol consumption [33]. The association of younger individuals is consistent with this study’s findings: <60 years of age is a significant factor associated with MDR-TB.

A study in Serbia in 2018 reported the risk factors for MDR-TB among 124 tuberculosis patients (31 cases and 93 controls), from September 2009 to June 2014 [39]. Six significant independent risk factors were identified including monthly family income, defaulting from treatment, stigma associated with TB, subjective feelings of sadness, use of sedatives and chronic obstructive pulmonary disease. Other general demographic factors, such as sex, age, education, occupation, and patient residence showed no significance [39]. A report in the Republic of Korea in 2020 found that the mean age, sex ratio, BMI symptoms, disease duration, and other CT findings were not significantly different between patients with MDR-TB (*n* = 90) and DS-TB (*n* = 90) [40]. A worldwide systematic review and meta-analyses reported that AFB smear positivity, lung cavity, a history of TB disease and a history of anti-TB treatment were the risk factors associated with MDR-TB [39]. However, the multivariate analysis revealed that other parameters, such as sex, age, residential area, education, marital status, occupation, diabetes mellitus, and HIV infection could not be used as independent predictors for MDR-TB [41].

Though there were limits on the number of patients and the lack of some parameters that may be related to MDR-TB, our study found that younger people were significantly associated with MDR-TB in upper northern Thailand. Here, we primarily analyzed the association of the key mutation profiles of the *rpoB* and *katG* genes with the patient’s factors. The statistical analysis using Chi-square revealed that there was no correlation between S531L *rpoB*-mutation and gender, age, nationality, province of residence, and the results of the AFB smear and DST (*p* > 0.05). However, older aged MDR-TB patients (≥60 years old) were found to be associated with the S315T *katG*-mutation (OR = 8.867, 95% CI 0.981–80.177, *p* = 0.023). Moreover, this study has updated the information on the genotypes of MDR-TB, related to the *rpoB* and *katG* genes and the *inhA* promoter. Three mutation patterns were initially reported in the Thai population in the upper northern region, highlighting the importance of monitoring and surveillance of MDR-TB genotypes. Further investigation shall be continued with larger samples and patient parameters to delineate the diversity and distribution of MDR-TB genotypes, as well as the risk factors for MDR-TB and key mutation profiles in upper northern Thailand.

## 4. Materials and Methods

### 4.1. Study Design, Study Period, and Ethics

This study was conducted from March 2015 to December 2019 at the Office of Disease Prevention and Control 1 (ODPC 1) and Chiang Rai Prachanukroh Hospital (CPH). ODPC 1 is responsible for all 8 provinces in the upper northern region of Thailand (Chiang Mai, Chiang Rai, Lamphun, Mae Hong Son, Lampang, Phrae, Nan and Phayao). CPH is located in Chiang Rai province in the north of the country. It is bordered by the Shan State of Myanmar to the north and Bokeo province of Laos to the east. The ethical issues of this study were approved by the Institutional Ethics Committee, Faculty of Associated Medical Sciences, Chiang Mai University, and Chiang Rai Prachanukroh Hospital (approval no.: AMSEC-62EM-024 and EC CRH 123/63 Ex).

### 4.2. Samples and Data Collection

The culture of clinical specimens using standard mycobacterial culture (2% Ogawa medium at 37 °C for 4–8 weeks) was performed at OPDC 1 and CPH. The standard drug susceptibility testing (DST) of isolates identified as belonging to the *M*. *tuberculosis* complex (MTBC) against first-line anti-TB agents was carried out via the proportion method. Isolates were identified as resistant if >1% colony growth occurred on Middlebrook 7H10 agar comprising the critical drug concentrations (0.2 µg/mL for INH, 1.0 µg/mL for RIF, 2.0 µg/mL for streptomycin (SM), and 5.0 µg/mL for ethambutol (EMB). Quality control was routinely performed during DST using the reference strain, *M. tuberculosis* (Mtb) H37Rv (ATCC 27294). MDR-TB was determined by the resistance of the corresponding Mtb isolate to at least RIF and INH [1]. DS-TB was considered when the Mtb isolated from TB patients was susceptible to all first-line anti-TB drugs (RIF, INH, SM and EMB).

DNA extraction of all MDR-Mtb isolates was conducted using the Anyplex™ II MTB/MDR Detection kit (see gene, the Republic of South Korea), according to the manufacturer’s instruction. Genomic DNA extracts were stored at −20 °C before use.

The inclusion criteria for data collection from patients included individuals living in northern Thailand, diagnosed with TB, and available laboratory results. Data from 42 MDR-TB cases (RIF and INH-resistant TB), and 68 DS-TB cases (RIF, INH, SM, and EMB susceptible TB) were collected at ODPC 1 and CPH. The collected data included gender, age, nationality, province of residence, the result of acid-fast bacilli (AFB) smear and drug susceptibility testing (DST).

### 4.3. DNA Sequencing and Analysis

Amplification of the *rpoB*, *katG*, and the *inhA* promoter region using primers covering 543 bp, 455 bp, and 455 bp of each gene was carried out by PCR [42]. Before sequencing, amplicons were purified with NucleoSpin^®^ Gel and PCR Clean-up (MACHEREY-NAGEL GmbH & Co. KG, Düren, Germany). The DNA sequencing was performed by Bioneer co., Ltd., using the ABI 3730XL DNA analyzer (Thermo Fisher Scientific, Waltham, MA, USA). The mutation profiles were analyzed using BioEdit Sequence Alignment version 7.2.5 [43]. The nucleotide sequences of the MDR-TB isolates were compared with reference strains *M*. *tuberculosis* H37Rv (NCBI Accession number: NC_000962.3). The mutation codon was identified based on the *Escherichia coli rpoB* gene codon.

### 4.4. Data Analysis

The comparative data analysis between DS-TB and MDR-TB was performed using descriptive statistics, chi-square, and Fisher’s exact test. The association of MDR-TB and S531L *rpoB* mutation, a key substitution mutation related to RIF resistance, was determined in comparison to other *rpoB* gene mutation profiles. Likewise, the association of MDR-TB and S315T *katG* mutation, a key mutation related to INH resistance, was also determined in comparison to other mutation profiles within the *katG* gene and the *inhA* promoter. The results of the analysis are considered significant when the *p*-value is less than 0.05. The analysis of all data was carried out using SPSS Software Version 17.0 (IBM, Armonk, NY, USA).

## Figures and Tables

**Table 1:** The frequency of the *rpoB*, *katG*, and *inhA* promoter mutations among 51 multidrug-resistant *Mycobacterium tuberculosis* isolates in the northern Thailand.

Mutation Profile		Frequency	Percent (%)
**The Mutation in *rpoB* Gene**			
***rpoB***	S531L (T**C**G>T**T**G)	26	50.98
	H526Y (**C**AC>**T**AC)	10	19.61
	H526D (**C**AC>**G**AC)	6	11.76
	D516V (G**A**C>G**T**C)	3	5.88
	S522L (T**C**G>T**T**G)	1	1.96
	H526R (C**A**C>C**G**C)	1	1.96
	H526P (C**A**C>C**C**C)	1	1.96
	Q513P (C**A**A>C**C**A)	1	1.96
	H526C (**CA**C>**TG**C)	1	1.96
	No mutation	1	1.96
**The mutation in *katG* and *inhA* promoter**			
***katG***	S315T (A**G**C>A**C**C)	27	52.94
***inhA*** **promoter**	−15 (C>T) −9 (T>C) −17 (G>T)	8 1 1	15.67 1.96 1.96
***katG/inhA*** **promoter**	S315T (A**G**C>A**C**C)/−8 (T>G) S315T (A**G**C>A**C**C)/−15 (C>T) S315T (A**G**C>A**C**C)/−17 (G>T)	3 1 1	5.88 1.96 1.96
	No mutation	9	17.67

**Table 2:** The genotype of 51 multidrug-resistant *Mycobacterium tuberculosis* isolates in upper northern Thailand.

Sample Code	Mutation Profile			Percent (%) (*n*)
	*rpoB*	*katG*	*inhA* Promoter	
MDR 1-5	S531L	No mutation	No mutation	9.80 (5)
MDR 6-16		S315T	No mutation	21.57 (11)
MDR 17-22		No mutation	−15 (C>T)	11.76 (6)
MDR 23		No mutation	−7 (G>T)	1.96 (1)
MDR 24-26		S315T	−8 (T>G)	5.88 (3)
MDR 27-29	H526Y	No mutation	No mutation	5.88 (3)
MDR 31-35		S315T	No mutation	11.76 (5)
MDR 36		No mutation	−15 (C>T)	1.96 (1)
MDR 37-41	H526D	S315T	No mutation	9.80 (5)
MDR 42		No mutation	−15 (C>T)	1.96 (1)
MDR 43-44	D516V	S315T	No mutation	3.92 (2)
MDR 45		No mutation	−9 (T>C)	1.96 (1)
MDR 46	S522L	S315T	−17 (G>T)	1.96 (1)
MDR 47	H526R	S315T	−15 (C>T)	1.96 (1)
MDR 48	Q513P	No mutation	No mutation	1.96 (1)
MDR 49	H526C	S315T	No mutation	1.96 (1)
MDR 50	H526P	S315T	No mutation	1.96 (1)
MDR 51	No mutation	S315T	No mutation	1.96 (1)

**Table 3:** The demographics and clinical characteristics among multidrug-resistant tuberculosis (MDR-TB) and drug-susceptible tuberculosis (DS-TB).

Characteristics (*n)*	MDR-TB (*n =* 42)	DS-TB (*n =* 68)	*p*-Value ^1^
**Gender**			
Male (78)	27 (64.29%)	51 (75.0%)	0.229
Female (32)	15 (35.71%)	17 (25.0%)	
**Age,** Mean ± S.D. (minimum-maximum)	48.3 ± 15.6 (23–89)	52.95 ±17.38 (17–84)	
<60 years (73)	34 (80.95%)	39 (57.35%)	**0.011** *
≥60 years (37)	8 (19.05%)	29 (42.65%)	
**Nationality**			
Thai (102)	38 (37.25%)	64 (90.12%)	0.478
Other (8)	4 (9.52%)	4 (5.88%)	
**Province of residence**			
Chiang Mai (35)	10 (23.81%)	25 (36.76%)	0.156
Other provinces (75)	32 (76.19%)	43 (63.24%)	
**AFB smear ^2^**			
Positive (83)	32 (84.21%)	51 (76.12%)	0.328
Negative (22)	6 (15.79%)	16 (23.88%)	
**Drug susceptibility testing**			
**Ethambutol**			
Resistant (2)	2 (4.76%%)	-	0.144
Susceptible (108)	40 (95.24%)	68 (100%)	
**Streptomycin**			
Resistant (9)	9 (21.24%)	-	1.000
Susceptible (101)	33 (78.57%)	68 (100%)	

^1^ *p*-value based on Chi-square test; * *p*-value less than 0.05. ^2^ AFB (acid-fast bacilli) smear result was not available in 5 tuberculosis cases (*n* = 105).

**Table 4:** Demographics and clinical characteristics of MDR-TB patients with different *rpoB* gene mutations (S531L MDR-TB vs. non-S531L MDR-TB).

Characteristics (*n*)	The *rpoB* Gene Mutation		*p*-Value ^1^
	S531L MDR-TB (*n* = 22)	Non-S531L MDR-TB (*n* = 20)	
**Gender**			
Male (27)	17 (77.27%)	10 (50.0%)	0.107
Female (15)	5 (22.73%)	10 (50.0%)	
**Age**	(44.8 ± 13.95) (24–75)	(52.2 ± 16.82) (23–89)	
<60 years (34)	20 (90.91%)	14 (70.0%)	0.123
≥60 years (8)	2 (9.09%)	6 (30.0%)	
**Nationality**			0.608
Thai (38)	19 (86.36%)	19 (95.0%)	
Other (4)	3 (13.64%)	1 (5.0%)	
**Province of Residence**			0.721
Chiang Rai (18)	10 (45.45%)	8 (40.0%)	
Other provinces (24)	12 (54.55%)	12 (60.0%)	
**Acid fast bacilli (AFB) ^2^**			0.868
Positive (32)	16 (80.0%)	16 (88.89%)	
Negative (6)	4 (20.0%)	2 (11.11%)	
**Drug susceptibility testing**			
**Ethambutol**			
Resistant (2)	1 (4.55%)	1 (5.0%)	0.945
Susceptible (40)	21 (95.45%)	19 (95.0%)	
**Streptomycin**			
Resistant (9)	5 (22.73%)	4 (20.0%)	0.830
Susceptible (33)	17 (77.27%)	16 (80.0%)	

^1^ *p*-value based on Chi-square test. ^2^ AFB (acid fast bacilli) smear result was not available in 4 tuberculosis cases (*n* = 38).

**Table 5:** Demographics and clinical characteristics of MDR-TB patients with different *katG* gene and *inhA* promoter mutations (S315T MDR-TB vs. non-S315T MDR-TB).

Characteristics (*n*)	The *katG* Gene and *inhA* Promoter Mutation		*p*-Value ^1^
	S315T MDR-TB (*n* =22)	Non-S315T MDR-TB (*n* =20)	
**Gender**			
Male (27)	14 (63.64%)	13 (65.0%)	0.927
Female (15)	8 (36.36%)	7 (35.0%)	
**Age**	(54.3 ± 16.4) (24–89)	(41.7 ± 11.9) (23–70)	
<60 years (34)	15 (68.18%)	19 (95.0%)	0.047 *
≥60 years (8)	7 (31.82%)	1 (5.0%)	
**Nationality**			
Thai (38)	20 (90.91%)	18 (90.0%)	1.000
Other (4)	2 (9.09%)	2 (10.0%)	
**Province of residence**			
Chiang Rai (18)	11 (50.0%)	7 (35.0%)	0.366
Other provinces (24)	11 (50.0%)	13 (65.0%)	
**Acid fast bacilli (AFB) ^2^**			
Positive (32)	16 (84.21%)	16 (84.21%)	0.635
Negative (6)	3 (15.79%)	3 (15.79%)	
**Drug susceptibility testing**			
**Ethambutol**			
Resistant (2)	1 (4.55%)	1 (5.0%)	0.945
Susceptible (40)	21 (95.45%)	19 (95.0%)	
**Streptomycin**			
Resistant (9)	7 (77.8%)	2 (10.0%)	0.135
Susceptible (33)	15 (57.7%)	18 (90.0%)	

^1^ *p*-value based on Chi-square test: * *p*-value less than 0.05. ^2^ AFB (acid fast bacilli) smear result was not available in 4 tuberculosis cases (*n* = 38).

## Data Availability

Not applicable.

## Supplementary Materials

The following supporting information can be downloaded at: https://www.mdpi.com/article/10.3390/antibiotics11121733/s1, Table S1: Genotypes and sources of 51 multidrug-resistant *Mycobacterium tuberculosis* isolates.
