# Supplementary material for: Genotypic Distribution and the Epidemiology of Multidrug Resistant Tuberculosis in Upper Northern Thailand
Source: Antibiotics (Basel). 2022 Dec 1;11(12):1733. doi: 10.3390/antibiotics11121733 (PMC9774302; doi:10.3390/antibiotics11121733)
Supplement: Supplementary file 1 [file antibiotics-11-01733-s001.zip › antibiotics-2037862-supplementary.pdf]

Table S1. Genotypes and sources of 51 multidrug-resistant *Mycobacterium tuberculosis* isolates.

Sample Code (n = 51)	Mutation			Province
	rpoB	*katG*	*inhA Promoter*	
MDR1	S531L	No mutation	No mutation	Chiang Mai
MDR2	S531L	No mutation	No mutation	Chiang Mai
MDR3	S531L	No mutation	No mutation	Chiang Mai
MDR4	S531L	No mutation	No mutation	Chiang Rai
MDR5	S531L	No mutation	No mutation	Chiang Rai
MDR6	S531L	S315T	No mutation	Lampang
MDR7	S531L	S315T	No mutation	Phayao
MDR8	S531L	S315T	No mutation	Nan
MDR9	S531L	S315T	No mutation	Nan
MDR10	S531L	S315T	No mutation	Phrae
MDR11	S531L	S315T	No mutation	Chiang Rai
MDR12	S531L	S315T	No mutation	Chiang Rai
MDR13	S531L	S315T	No mutation	Chiang Rai
MDR14	S531L	S315T	No mutation	Chiang Rai
MDR15	S531L	S315T	No mutation	Chiang Rai
MDR16	S531L	S315T	No mutation	Chiang Mai
MDR17	S531L	No mutation	-15	Nan
MDR18	S531L	No mutation	-15	Chiang Mai
MDR19	S531L	No mutation	-15	Chiang Rai
MDR20	S531L	No mutation	-15	Chiang Rai
MDR21	S531L	No mutation	-15	Chiang Rai
MDR22	S531L	No mutation	-15	Chiang Rai
MDR23	S531L	No mutation	-17	Chiang Mai
MDR24	S531L	S315T	-8	Lamphun
MDR25	S531L	S315T	-8	Lamphun
MDR26	S531L	S315T	-8	Lamphun
MDR27	H526Y	No mutation	No mutation	Chiang Mai
MDR28	H526Y	No mutation	No mutation	Chiang Mai
MDR29	H526Y	No mutation	No mutation	Chiang Rai
MDR30	H526Y	S315T	No mutation	Chiang Mai
MDR31	H526Y	S315T	No mutation	Lampang
MDR32	H526Y	S315T	No mutation	Chiang Mai
MDR33	H526Y	S315T	No mutation	Lampang
MDR34	H526Y	S315T	No mutation	Lampang
MDR35	H526Y	S315T	No mutation	Chiang Mai
MDR36	H526Y	No mutation	-15	Lampang
MDR37	H526D	S315T	No mutation	Lampang
MDR38	H526D	S315T	No mutation	Phrae
MDR39	H526D	S315T	No mutation	Chiang Rai
MDR40	H526D	S315T	No mutation	Chiang Rai
MDR41	H526D	S315T	No mutation	Chiang Rai
MDR42	H526D	No mutation	-15	Phrae
MDR43	D516V	S315T	No mutation	Chiang Rai
MDR44	D516V	S315T	No mutation	Phayao
MDR45	D516V	No mutation	-9	Chiang Mai
MDR46	S522L	S315T	-17	Chiang Mai
MDR47	H526R	S315T	-15	Lampang
MDR48	Q513P	No mutation	No mutation	Chiang Rai
MDR49	H526C	S315T	No mutation	Chiang Rai
MDR50	H526P	S315T	No mutation	Chiang Rai
MDR51	No mutation	S315T	No mutation	Lampang
